# Trauma Erodes Financial Returns of Educational Attainment

**DOI:** 10.31586/ojer.2025.1199

**Published:** 2025-02-14

**Authors:** Shervin Assari, Alexandra Donovan

**Affiliations:** 1 Department of Internal Medicine, College of Medicine, Charles R. Drew University of Medicine and Science, 1731 E. 120th St., Los Angeles, CA, 90059, USA; 2 Department of Family Medicine, College of Medicine, Charles R. Drew University of Medicine and Science, 1731 E. 120th St., Los Angeles, CA, 90059, USA; 3 Department of Public Health, Charles R. Drew University of Medicine and Science, 1731 E. 120th St., Los Angeles, CA, 90059, USA

**Keywords:** Minorities’ Diminished Returns, MDRs, Education, Income-to-Needs Ratio, Stress, Trauma, Socioeconomic Disparities, Economic Inequality, Racial Disparities, Structural Inequities, National Survey of American Life, Chronic Stress, Marginalized Populations, Lifetime Trauma

## Abstract

**Background:**

Educational attainment is often regarded as a pathway to economic stability and social mobility. However, the Minorities’ Diminished Returns (MDRs) framework has demonstrated that the effects of educational attainment on various economic, behavioral, and health outcomes are weaker for marginalized populations, including racial/ethnic minorities, immigrants, LGBTQ+ individuals, and those living in disadvantaged areas. While MDRs have been documented for various marginalized demographic groups, the role of trauma in moderating socioeconomic outcomes remains underexplored.

**Objective:**

This study examines whether lifetime trauma exposure diminishes the positive association between educational attainment and poverty-to-income ratio (PIR), a key indicator of economic well-being.

**Methods:**

Using data from the National Survey of American Life (NSAL), we analyzed a nationally representative sample of 6,008 adults, including Black, White, Latino, and Other racial/ethnic groups. We employed linear regression models to evaluate the association between the independent variable educational attainment and the outcome PIR. We then tested lifetime trauma as a moderator of this association. Models controlled for age, gender, employment, and race/ethnicity.

**Results:**

Educational attainment was positively associated with PIR across all groups, but the strength of this association was significantly attenuated for individuals with a history of lifetime trauma. These effects were independent of covariates.

**Conclusions:**

These findings extend the MDRs framework by highlighting trauma as a potential contributor to diminished returns of education on socioeconomic well-being. Structural inequities that increase trauma exposure in minoritized populations may also limit the economic benefits of education, particularly for groups with multiple trauma exposures. Policies aimed at addressing economic inequality must integrate social policies that reduce trauma and stress.

## 1. Introduction

Educational attainment has long been recognized as a cornerstone of economic stability and social advancement [1,2]. In theory, higher levels of educational attainment equip individuals with the skills and opportunities necessary to secure stable employment, achieve financial independence, and improve their quality of life [3–5]. For decades, policymakers have promoted educational attainment as a universal tool for reducing poverty and inequality [6–13]. However, growing evidence suggests that the economic benefits of educational attainment are not equally distributed across all populations [10,11,14,15].

The concept of Minorities’ Diminished Returns (MDRs) [16,17] offers a critical framework for understanding these disparities. MDRs propose that marginalized groups, including racial [18–25] and ethnic [18,26–29] minorities, immigrants [30–38], LGBTQ+ [39,40] individuals, and those residing in socioeconomically disadvantaged areas [41] derive weaker benefits from socioeconomic resources such as educational attainment and income. One plausible and empirically supported mechanism for MDRs among Black [20–22,42–44], Latino [45–49], Asian [50,51], Native American [52–54], LGBTQ+ [39,40], and immigrant [30–32,34,36,38] populations is their disproportionate exposure to chronic stressors and traumatic experiences [55–57]. These stressors, which include systemic discrimination, social exclusion, and higher rates of violence or adversity, can impair individuals’ ability to fully capitalize on their educational achievements, undermining the effectiveness of traditional pathways to success [17].

Lifetime trauma—such as experiences of violence, abuse, or natural disasters—is a pervasive and chronic stressor that can disrupt cognitive, emotional, and social functioning [58–61]. These skills are critical for translating educational attaingment into tangible economic benefits such as employment, financial management, and long-term stability. For instance, trauma can lead to long-term psychological effects such as anxiety, depression, and post-traumatic stress disorder (PTSD), which reduce employability by impairing concentration, decision-making, and interpersonal skills. Moreover, trauma-induced loss of social networks or family support may limit access to job opportunities and mentorship [62–64]. Individuals who experience trauma often face challenges in managing workplace stress, maintaining professional relationships, or meeting the demands of high-performance environments—prerequisites for career advancement [65]. Gaps in employment history due to trauma-related health problems or caregiving responsibilities can further stigmatize job seekers, making it harder for even highly educated individuals to compete in the labor market [66–69]. These examples illustrate how trauma and stress can undermine the economic returns of education, thereby contributing to MDRs in marginalized populations.

While the effects of MDRs have been well-documented across race [18–25], ethnicity [18,26–29], nativity [30–38], sexual orientation [39,40], and place [41], less attention has been devoted to the role of trauma in shaping these dynamics. Exposure to trauma is not only common but also relatively easy to measure using standardized tools and self-reported assessments. Its pervasiveness and quantifiable characteristics make trauma an accessible and important variable for research across disciplines [70,71]. Trauma is extensively documented as a predictor of numerous health problems, including mental health disorders such as depression and anxiety [72], and physical conditions such as cardiovascular disease and chronic pain [73]. However, its potential to weaken the socioeconomic benefits of educational attainment remains underexplored. By impairing resilience and reducing productivity, trauma may act as a hidden moderator that reduces an individuals’ ability to fully leverage their education [66]. Investigating trauma as a factor in MDRs offers a critical opportunity to understand how stress and adversity undermine the effects of educational attainment on economic well-being.

This study seeks to expand the MDRs framework [40,47,74–81] by examining whether lifetime trauma diminishes the positive association between educational attainment and economic well-being. Using data from the National Survey of American Life (NSAL) [82–84], we test two hypotheses: first, that higher educational attainment is associated with a higher poverty-to-income ratio (PIR); and second, that this positive effect is weaker for individuals who have experienced trauma, after accounting for gender, age, employment, and racial/ethnic minority status. This research aims to advance understanding of the interplay between education, trauma, and economic inequality.

## 2. Methods

### 2.1. Study Design and Sample

The National Survey of American Life (NSAL) [82–84] is a nationally representative survey conducted in 2003, designed to investigate the mental health, socioeconomic conditions, and social environments of the U.S. population, with an emphasis on Black and Caribbean communities. The NSAL included 6,082 participants, consisting of Black, White, Latino, and Other racial/ethnic groups. This study focuses on a subset of 6,008 individuals with complete data on key variables, including educational attainment, poverty-to-income ratio (PIR), and lifetime trauma. The NSAL employed a multi-stage area probability sampling design to ensure representation of diverse racial/ethnic groups across the United States. Black and Caribbean populations were intentionally oversampled to provide robust data on these groups, making the NSAL uniquely suited for studying the intersections of race, trauma, and socioeconomic outcomes.

### 2.2. Analytical sample

This study included all participants from the National Survey of American Life (NSAL) who had complete data on lifetime trauma exposure, educational attainment, poverty-to-income ratio (PIR), and relevant covariates. The inclusion criteria were non-restrictive, allowing individuals from all racial and ethnic backgrounds to be part of the analysis, provided they reported data on the variables of interest. Trauma exposure was assessed through a standardized checklist of lifetime events, educational attainment was measured in years of schooling completed, and PIR was calculated as a ratio of household income to the federal poverty threshold. Covariates such as age, gender, marital status, and employment status were included to control for potential confounders in the relationship between education, trauma, and economic outcomes. This inclusive approach ensures that the findings are applicable across diverse populations and provides a comprehensive understanding of how trauma moderates the returns of educational attainment on economic well-being.

### 2.3. Variables

#### 2.3.1. Outcome Variable:

##### Poverty-to-Income Ratio (PIR):

PIR was calculated as the ratio of household income to the federal poverty threshold, with values greater than 1 indicating income above the poverty line and values below 1 indicating income below the poverty line. PIR was treated as a continuous variable, capturing economic well-being and financial stability.

#### 2.3.2. Predictor Variable:

##### Educational Attainment:

Educational attainment was assessed as the total years of schooling completed, measured as a continuous variable in primary analyses. Educational attainment was then categorized into three levels: less than high school, high school graduate, some college, and bachelor’s degree or higher.

#### 2.3.3. Moderator Variable:

##### Lifetime Trauma:

Trauma exposure was assessed using a checklist of lifetime traumatic events including: (1) health problems, (2) financial problems, (3) job-related issues, (4) family or marital problems, (5) being a victim of a crime, (6) conflicts with law enforcement, (7) experiences of racial discrimination, and (8) difficulties with gambling. For respondents who were parents, an additional item (9) addressed problems with children. Since item 9 was irrelevant for individuals without children, we calculated the total trauma score as the average of all valid items, ensuring comparability between parents and non-parents. This approach provided a standardized measure of trauma exposure with a potential range from 0 to 1, where higher scores indicated greater levels of stress over the lifetime. To facilitate analysis, we also created a categorical measure to classify individuals into one of three groups: no stress, one stressor, or two or more stressors. This measure has been previously validated and widely used in analyses of the NSAL dataset [56,85].

#### 2.3.4. Covariates:

##### Age:

Measured in years and treated as a continuous variable.

##### Gender:

Coded as male or female (reference group).

##### Marital Status:

Categorized as married vs unmarried (divorced, separated, widowed, or never married). Unmarried was the reference group.

##### Employment Status:

Coded as employed (full-time or part-time) or unemployed (reference group).

##### Race/Ethnicity:

Self-reported race/ethnicity was categorized as African American, Caribbean Black, or Non-Latino White (reference group).

### 2.4. Statistical Analysis

A series of linear regression models were used to evaluate the association between educational attainment and PIR, with lifetime trauma included as a moderator. Interaction terms were added to test whether trauma moderates the relationship between educational attainment and PIR. The analysis proceeded as follows:

#### Model 1:

Examined the main effect of educational attainment on PIR, adjusting for age, gender, marital status, employment status, and race/ethnicity.

#### Model 2:

Added lifetime trauma as a main effect to evaluate its independent association with PIR.

#### Model 3:

Included an interaction term between educational attainment and lifetime trauma to test whether trauma moderates the effect of educational attainment on PIR.

#### Stratified Analyses:

Models were stratified by number of traumas to explore potential variations in the education-PIR association and its moderation by trauma.

To account for the complex sampling design of the National Survey of American Life (NSAL), all analyses incorporated survey weights to ensure that estimates were representative of the U.S. population. The NSAL utilized a multi-stage area probability sampling method, which involved stratification and clustering to achieve a nationally representative sample with an oversample of Black and Caribbean populations. Survey weights were applied to adjust for unequal probabilities of selection, nonresponse, and post-stratification, ensuring that results could be generalized to the broader population. Additionally, the design effects of clustering and stratification were accounted for by estimating robust standard errors using Taylor series linearization. This method provides accurate variance estimates and ensures the validity of statistical inferences. By integrating survey weights and correctly estimating standard errors, this study maintains methodological rigor and accounts for potential biases introduced by the sampling design.

Statistical significance was determined at a two-tailed alpha level of 0.05. From linear regressions, we reported B, SE, 95% CI, and p values. All statistical analyses were conducted using Stata/SE 18.0, which accommodates survey weighting and clustering to provide accurate estimates and standard errors. Visualizations of interaction effects were created to aid interpretation of results and highlight variations in PIR by educational attainment and trauma status.

## 3 . Results

Table 1 summarizes the results of three models analyzing the association between demographic factors, educational attainment, trauma exposure, and poverty-to-income ratio (PIR) in the pooled sample.

Model 1 shows the baseline association of demographic factors and educational attainment with PIR. Males had significantly higher PIRs than females (B = 0.604, SE = 0.109, p < 0.001), and older age was associated with a modest but significant increase in PIR (B = 0.015, SE = 0.004, p = 0.001). African Americans had significantly lower PIRs compared to non-Latino Whites (B = −0.448, SE = 0.152, p = 0.004), while differences for Latinos and Caribbean Blacks were not significant. Being married was strongly associated with a higher PIR (B = 1.031, SE = 0.107, p < 0.001), while being unemployed was associated with a significantly lower PIR (B = −0.652, SE = 0.311, p = 0.040). Educational attainment demonstrated a clear gradient, with higher levels of education associated with progressively higher PIRs. For instance, those with 12 years (B = 0.796, SE = 0.097, p < 0.001), 13–15 years (B = 1.401, SE = 0.157, p < 0.001), and 16+ years of education (B = 2.874, SE = 0.231, p < 0.001) all had significantly higher PIRs compared to those with 11 years or less.

Model 2 introduces trauma exposure as a predictor. Males (B = 0.563, SE = 0.099, p < 0.001) and married individuals (B = 0.974, SE = 0.105, p < 0.001) continued to show significantly higher PIRs, while African Americans still exhibited lower PIRs compared to non-Latino Whites (B = −0.360, SE = 0.152, p = 0.020). Trauma exposure was inversely associated with PIR, with individuals reporting one traumatic event (B = −0.546, SE = 0.136, p < 0.001) and two or more traumatic events (B = −0.882, SE = 0.115, p < 0.001) having progressively lower PIRs compared to those with no trauma. Educational attainment remained a strong predictor, with higher levels of education associated with greater PIR. The association persisted for 12 years (B = 0.733, SE = 0.111, p < 0.001), 13–15 years (B = 1.271, SE = 0.138, p < 0.001), and 16+ years of education (B = 2.717, SE = 0.217, p < 0.001).

Model 3 examines the interaction between trauma exposure and educational attainment. The main effects of gender, age, marital status, and African American race were consistent with earlier models. While trauma exposure alone (one or two events) did not show significant associations in this model, the interaction terms revealed significant moderation of education’s effect on PIR by trauma. Specifically, among individuals with one traumatic event, the association between 13–15 years of education and PIR was significantly attenuated (B = −0.531, SE = 0.229, p = 0.024). Among individuals with two or more traumatic events, the diminished returns of education were even more pronounced, with significant reductions in PIR for 13–15 years (B = −1.033, SE = 0.244, p < 0.001) and 16+ years (B = −1.775, SE = 0.647, p = 0.008) of education compared to individuals with no trauma.

These results suggest that while educational attainment is a strong predictor of higher PIR in the general population, its benefits are significantly diminished for individuals with trauma exposure, particularly those with multiple traumatic events. This highlights the role of trauma as a critical moderator of socioeconomic outcomes.

Table 2 presents the results of stratified regression models examining the association between demographic factors, educational attainment, and poverty-to-income ratio (PIR) across three levels of trauma exposure (0, 1, and 2+ lifetime stressors). Among individuals with no reported trauma, males had significantly higher PIRs compared to females (B = 0.586, SE = 0.129, p < 0.001), and older age was associated with a small but significant increase in PIR (B = 0.017, SE = 0.006, p = 0.005). Being married was strongly associated with higher PIR (B = 1.131, SE = 0.159, p < 0.001), while unemployment was not significant. Educational attainment showed a clear gradient, with those completing 12 years (B = 1.039, SE = 0.169, p < 0.001), 13–15 years (B = 1.765, SE = 0.188, p < 0.001), and 16+ years of education (B = 3.059, SE = 0.282, p < 0.001) having progressively higher PIRs compared to those with 11 or fewer years. Race/ethnicity differences were not statistically significant in this group.

For individuals with one reported trauma, males continued to have higher PIRs (B = 0.560, SE = 0.158, p = 0.001), but the effect of age was no longer significant. African Americans in this group had significantly lower PIRs compared to non-Latino Whites (B = −0.604, SE = 0.227, p = 0.010), while Latino and Caribbean Black groups showed no significant differences. Being married remained positively associated with PIR (B = 0.856, SE = 0.106, p < 0.001), but unemployment showed a marginal negative association (B = −0.495, SE = 0.271, p = 0.072). Educational attainment maintained a strong association with PIR, with 12 years (B = 0.523, SE = 0.222, p = 0.021), 13–15 years (B = 1.071, SE = 0.196, p < 0.001), and 16+ years (B = 2.935, SE = 0.343, p < 0.001) associated with progressively higher PIRs compared to 11 years or less.

Among those with two or more reported traumas, the positive association between being male and PIR was no longer statistically significant (B = 0.634, SE = 0.375, p = 0.096), and age remained non-significant. Race/ethnicity differences were also non-significant in this group. Marriage was positively associated with PIR (B = 0.629, SE = 0.204, p = 0.003), while unemployment was strongly associated with lower PIR (B = −0.757, SE = 0.227, p = 0.001). Educational attainment continued to show a significant gradient, though the magnitude of the associations decreased compared to the other groups, with 12 years (B = 0.725, SE = 0.262, p = 0.007), 13–15 years (B = 0.620, SE = 0.217, p = 0.006), and 16+ years (B = 1.227, SE = 0.485, p = 0.014) still associated with higher PIRs compared to those with 11 years or less.

Overall, the results indicate that educational attainment is a consistent predictor of PIR, though its magnitude diminishes as trauma exposure increases. Other demographic factors, such as gender, marital status, and employment, also interact differently with PIR depending on the level of trauma exposure.

## 4. Discussion

The aim of this study is twofold. First, the study seeks to examine the main effect of educational attainment on poverty-to-income ratio, hypothesizing that higher educational attainment will be associated with a higher poverty-to-income ratio across the entire sample. This reflects the general understanding that educational attainment provides individuals with the skills and opportunities needed to improve their economic well-being. Second, the study aims to investigate the variation in this relationship based on lifetime stress exposure. Specifically, the hypothesis is that the positive association between educational attainment and poverty-to-income ratio will be weaker among individuals who have experienced lifetime stress compared to those without such exposure. This second aim is grounded in the framework of Minorities’ Diminished Returns (MDRs), which suggests that stress may act as a barrier that limits the full benefits of educational attainment by impairing cognitive, emotional, and social functioning, and by exacerbating structural inequities that disproportionately impact stress-exposed individuals. Together, these aims address both the effects of educational attainment on economic outcomes and the conditions under which these effects are diminished.

This study provides additional evidence that lifetime trauma moderates the relationship between educational attainment and economic well-being, consistent with the framework of Minorities’ Diminished Returns (MDRs) [16]. Higher levels of educational attainment were associated with higher PIR across the sample, but this association was significantly weaker among individuals with a history of trauma. This was net of controlling for race/ethnicity, and other covariates.

Trauma is not the only factor that reduces the returns of education. Various marginalized identities [16] are shown to reduce the returns of educational attainment. These include racial and ethnic minorities like Black [14,86–89], Latino [28,29,90,91], Asian [51,52,54,92], and American Indian/Alaska Native (AIAN) [8,59,61,90] populations, who face systemic barriers that reduce the benefits of educational and economic attainment. Immigrants [30–34,36,38] and LGBTQ+ [39,40] individuals similarly experience diminished returns due to discrimination, social exclusion, and structural inequities. Marginalized White populations, particularly those in low-income or rural areas, also show evidence of MDRs, as limited opportunities and geographic inequities constrain their ability to capitalize on resources. A key factor underlying these diminished returns across diverse groups is chronic and cumulative stress. Marginalized individuals often endure higher levels of stress due to racism, stigma, microaggressions, and economic instability, which disrupts the biological and psychological processes necessary for fully leveraging socioeconomic advantages. This stress undermines productivity, resilience, and access to opportunities, making it a crucial driver of MDRs across varied social identities.

The phenomenon of Minorities’ Diminished Returns (MDRs) [17] arises from multiple interrelated causes, many of which reflect the structural, environmental, and psychosocial inequities faced by marginalized populations. One primary driver is chronic stress, which results from cumulative exposure to discrimination, stigma, and economic instability [57,93]. Chronic stress can dysregulate biological systems, impair cognitive functioning, and reduce the capacity to capitalize on resources such as education. Structural barriers, including labor market discrimination, limited access to high-paying jobs, and residential segregation, further erode the potential benefits of socioeconomic resources. Institutional racism and unequal policies systematically devalue the educational attainment and income of marginalized groups, limiting their upward mobility. Cultural and social factors, such as reduced access to supportive networks or exclusion from elite institutions, also contribute by curtailing opportunities for growth. Finally, health disparities, including higher rates of physical and mental health conditions among marginalized populations, may reduce productivity and earning potential, further weakening the returns on educational attainment and other socioeconomic assets. Together, these mechanisms highlight the complex and layered processes that constrain the effectiveness of socioeconomic resources in reducing inequality for marginalized groups.

Lifetime trauma may operate as a critical moderator of socioeconomic outcomes [66,68]. Trauma, as a chronic stressor, undermines the ability of individuals to translate educational attainment into financial stability, likely by interfering with employment opportunities, cognitive function, and social networks [94–97]. This study highlights the intersection of structural and psychosocial factors in perpetuating economic disparities.

### 4.1. Implications

The findings underscore the need for policies that address the compounding effects of structural inequities and trauma by enhancing the economic resilience of trauma-exposed individuals. While trauma-informed care and trauma screening have become critical components of health and social services, there is a growing need to extend these principles to trauma-informed policymaking. Such an approach would involve designing policies and programs that explicitly acknowledge and address the long-term impacts of trauma on individuals’ ability to mobilize their education and generate income. Trauma-informed policymaking would prioritize creating supportive environments in workplaces, schools, and communities to reduce barriers faced by those who have experienced trauma. For example, workforce development programs could integrate mental health support, flexible work arrangements, and targeted mentorship opportunities to help trauma survivors overcome employment challenges. Education policies could incorporate trauma-informed teaching practices, ensuring that students affected by adversity have the resources and support to succeed academically and transition into the labor market effectively. Economic policies, such as enhanced access to financial assistance, affordable housing, and childcare, could alleviate chronic stressors that disproportionately affect trauma-exposed individuals, enabling them to better utilize their educational qualifications. By embedding trauma-informed principles into policy frameworks, we can create systemic solutions that help individuals leverage their education to achieve economic stability and mobility, reducing disparities and fostering resilience in marginalized populations.

### 4.2. Limitations

This study has several limitations. The cross-sectional design precludes causal inferences about the relationship between education, trauma, and economic outcomes. Data were collected about 20 years ago and COVID pandemic, inflation, and political changes may have changed economic well-being of populations as well as exposure to trauma, limiting applicability to current populations. Self-reported measures of trauma may be subject to recall bias, and the NSAL’s trauma checklist does not capture the full complexity of traumatic experiences. In addition, the study focuses on a single economic outcome (PIR), which may not capture other dimensions of financial well-being. Finally, the study did not test the role of marginalizing identities on reducing the returns of educational attainment on PIR.

### 4.3. Future Directions

Future research should explore longitudinal trajectories of economic well-being among trauma-exposed individuals, examining how educational attainment interacts with trauma over time. Expanding the MDRs framework to include additional moderators, such as mental health and community resources, would further elucidate the mechanisms underlying these disparities. Finally, analysis of more recent data would allow for the study of the effects of any trauma-informed policies enacted within the last 20 years.

## 5. Conclusion

Lifetime trauma moderates the effects of educational attainment on economic well-being, reinforcing the need for integrated approaches to reducing economic disparities, especially among minoritized groups. Addressing trauma as part of broader equity initiatives is essential to ensuring that educational attainment serves as a pathway to opportunity for all individuals, regardless of their background or experiences.

## Figures and Tables

**Table 1.** Summary of Models in the Pooled Sample

	B	SE	95%	CI	p
**Model 1**					
Male	0.604	0.109	0.386	0.821	< 0.001
Age	0.015	0.004	0.006	0.023	0.001
Race/ethnicity					
Non-Latino White	Ref				
Latino	0.264	0.709	−1.151	1.679	0.711
Caribbean Black	−0.118	0.199	−0.515	0.279	0.555
African American	−0.448	0.152	−0.752	−0.144	0.004
Married	1.031	0.107	0.818	1.244	< 0.001
Unemployed	−0.652	0.311	−1.271	−0.032	0.040
Education					
11 Years or Less	Ref				
12 Years	0.796	0.097	0.602	0.991	< 0.001
13–15 Years	1.401	0.157	1.087	1.715	< 0.001
16+ Years	2.874	0.231	2.414	3.334	< 0.001

**Model 2**					
Male	0.563	0.099	0.367	0.760	0.000
Age	0.012	0.004	0.003	0.020	0.012
Race/ethnicity					
Non-Latino White	Ref				
Latino	0.320	0.761	−1.199	1.839	0.676
Caribbean Black	0.009	0.199	−0.389	0.407	0.964
African American	−0.360	0.152	−0.662	−0.057	0.020
Married	0.974	0.105	0.764	1.183	< 0.001
Unemployed	−0.401	0.291	−0.982	0.181	0.174
Trauma					
0	Ref				
1	−0.546	0.136	−0.816	−0.275	< 0.001
2+	−0.882	0.115	−1.111	−0.652	< 0.001
Education					
11 Years or Less	Ref				
12 Years	0.733	0.111	0.511	0.955	< 0.001
13–15 Years	1.271	0.138	0.995	1.547	< 0.001
16+ Years	2.717	0.217	2.284	3.151	< 0.001

**Model 3**					
Male	0.560	0.100	0.361	0.758	< 0.001
Age	0.012	0.004	0.003	0.021	0.008
Race/ethnicity					
Non-Latino White	Ref				
Latino	0.274	0.739	−1.200	1.749	0.712
Caribbean Black	0.010	0.208	−0.405	0.425	0.962
African American	−0.376	0.165	−0.705	−0.047	0.026
Married	0.940	0.094	0.752	1.129	< 0.001
Unemployed	−0.422	0.297	−1.014	0.170	0.159
Trauma					
0	Ref				
1	−0.247	0.259	−0.764	0.271	0.345
2+	−0.265	0.208	−0.680	0.149	0.205
Education					
11 Years or Less	Ref				
12 Years	0.990	0.172	0.646	1.334	< 0.001
13–15 Years	1.681	0.188	1.305	2.057	< 0.001
16+ Years	2.998	0.267	2.466	3.531	< 0.001
Trauma × Education					
1 × 12 Years	−0.419	0.307	−1.032	0.194	0.177
1 × 13–15 Years	−0.531	0.229	−0.988	−0.073	0.024
1 × 16+ Years	0.036	0.395	−0.751	0.824	0.927
2+ × 12 Years	−0.251	0.277	−0.803	0.301	0.368
2+ × 13–15 Years	−1.033	0.244	−1.521	−0.545	< 0.001
2+ × 16+ Years	−1.775	0.647	−3.066	−0.484	0.008

Note: Outcome: POVINDEX (poverty-to-income ratio [PIR]; higher score, higher socioeconomic status)

**Table 2.** Summary of Stratified Models

	B	SE	95%	CI	p
**Trauma= 0**					
Male	0.586	0.129	0.329	0.843	< 0.001
Age	0.017	0.006	0.005	0.030	0.005
Race/ethnicity					
Non-Latino White	Ref				
Latino	−0.300	0.300	−0.897	0.298	0.320
Caribbean Black	0.287	0.326	−0.363	0.936	0.382
African American	−0.274	0.244	−0.761	0.213	0.266
Married	1.131	0.159	0.814	1.449	< 0.001
Unemployed	0.449	0.865	−1.277	2.176	0.605
Education					
11 Years or Less	Ref				
12 Years	1.039	0.169	0.701	1.376	< 0.001
13–15 Years	1.765	0.188	1.390	2.141	< 0.001
16+ Years	3.059	0.282	2.496	3.622	< 0.001

**Trauma= 1**					
Male	0.560	0.158	0.246	0.875	0.001
Age	0.008	0.005	−0.003	0.019	0.141
Race/ethnicity					
Non-Latino White	Ref				
Latino	0.972	1.469	−1.959	3.903	0.510
Caribbean Black	−0.224	0.316	−0.854	0.406	0.481
African American	−0.604	0.227	−1.056	−0.151	0.010
Married	0.856	0.106	0.645	1.067	< 0.001
Unemployed	−0.495	0.271	−1.035	0.045	0.072
Education					
11 Years or Less					
12 Years	0.523	0.222	0.081	0.965	0.021
13–15 Years	1.071	0.196	0.680	1.461	< 0.001
16+ Years	2.935	0.343	2.251	3.619	< 0.001

**Trauma= 2+**					
Male	0.634	0.375	−0.114	1.381	0.096
Age	0.003	0.007	−0.011	0.017	0.638
Race/ethnicity					
Non-Latino White	Ref				
Latino	0.110	0.243	−0.375	0.594	0.653
Caribbean Black	−0.063	0.276	−0.612	0.487	0.821
African American	−0.105	0.184	−0.471	0.262	0.571
Married	0.629	0.204	0.221	1.037	0.003
Unemployed	−0.757	0.227	−1.210	−0.303	0.001
Education					
11 Years or Less	Ref				
12 Years	0.725	0.262	0.202	1.249	0.007
13–15 Years	0.620	0.217	0.188	1.052	0.006
16+ Years	1.227	0.485	0.259	2.194	0.014

Note: Outcome: POVINDEX (poverty-to-income ratio [PIR]; higher score, higher socioeconomic status)
